# Clinical significance of MRI-measured olfactory bulb height as an imaging biomarker of idiopathic Parkinson’s disease

**DOI:** 10.1371/journal.pone.0312728

**Published:** 2024-10-28

**Authors:** Sohyun Park, Jungbin Lee, Jung Youn Kim, Jeong-Ho Park, Ji Eun Lee, Sang Ik Park, Younghee Yim

**Affiliations:** 1 Department of Radiology, Soonchunhyang University Bucheon Hospital, Soonchunhyang University College of Medicine, Bucheon, Republic of Korea; 2 Department of Radiology, Chung-Ang University Hospital, Chung-Ang University College of Medicine, Seoul, Republic of Korea; 3 Department of Radiology, CHA University Bundang Medical Center, Seongnam, Republic of Korea; 4 Department of Neurology, Soonchunhyang University Bucheon Hospital, Bucheon, Republic of Korea; Clinical Investigation Center, TUNISIA

## Abstract

**Objectives:**

This study aimed to determine whether the olfactory bulb height (OBH) measured using magnetic resonance imaging (MRI) has clinical utility as an imaging biomarker in the evaluation of patients with idiopathic Parkinson’s disease (iPD) through its correlation with movement impairment.

**Methods:**

This retrospective study included cognitively intact patients with suspected parkinsonism. All participants underwent T2-weighted imaging to measure OBH. Logistic regression was used to determine whether OBH was an independent risk factor for distinguishing iPD patients from disease controls, and its relation with clinical parameters related to motor impairment, including clinical laterality, modified Hoehn and Yahr (HY) stage, and Unified Parkinson’s Disease Rating Scale (UPDRS) III score, was investigated.

**Results:**

Based on the final clinical diagnosis, 79 patients with iPD and 16 disease controls were included. The mean OBH was significantly smaller in iPD than in disease controls (*p* < 0.0001). OBH was a significant independent predictor of iPD, with a cutoff of 1.52 mm. In the comparison among the ipsilateral, contralateral side of iPD with clinical laterality, and disease control group, the OBH of the disease control group was significantly larger than both the ipsilateral and contralateral sides (*P* < 0.05). However, there was no significant difference in OBH between the ipsilateral and contralateral sides (*p* > 0.05). OBH according to HY stage was significantly smaller in HY stage 2–3 groups than in the disease control group (*p* < 0.001). The correlation analysis between UPDRS III and OBH showed a mild negative correlation (r = -0.32, *p* = 0.013).

**Conclusions:**

MRI-measured OBH is decreased in iPD regardless of age and sex and may be correlated with the progression of motor symptoms in the iPD.

## Introduction

Recently, smell has become an interesting topic in neurodegenerative disorder research. In particular, loss of olfaction in idiopathic Parkinson’s disease (iPD) has been a well-known feature since it was first reported in 1975 [1]. According to a multicenter study, olfactory dysfunction occurs in more than 95% of iPD cases [2]. Although the mechanism by which olfactory dysfunction occurs in iPD is not yet fully understood, both peripheral and central olfactory pathways appear to be involved. Braak et al. [3] hypothesized that Lewy pathology occurs in the olfactory bulb in the early stages. Many studies have investigated the usefulness of various clinical olfactory tests for diagnosing iPD and assessing disease progression [4]. However, because loss of smell is a subjective symptom, clinical olfactory function tests do not completely reflect the actual degree of olfactory dysfunction and are limited by the influence of the patient’s cognition [5]. In addition, iPD patients often subjectively overestimate their olfactory ability, making olfactory function tests difficult to perform clinically [6].

Accordingly, attempts have been made to measure the olfactory bulb using magnetic resonance imaging (MRI) [7–9]. Because parkinsonism evaluation often includes brain MRI, obtaining images to evaluate the olfactory bulb is simpler than a clinical olfactory function test and the data is relatively objective. However, most previous studies have compared healthy controls and patients with iPD, which is different from the clinical situation where an MRI is performed for the evaluation of parkinsonism. While right and left measurements have been described for each pair of olfactory bulbs, there are few results on the relationship with the laterality of motor dysfunction observed in iPD. Furthermore, few studies have investigated the correlation between motor symptoms and olfactory bulb height (OBH). Because there is still an unmet clinical need for tools to evaluate disease progression in iPD [10], a correlation between olfactory bulb measurement and motor disability in iPD, which can be quantitatively assessed, may add value to the evaluation of iPD. With recent advances in MRI techniques, research on olfactory bulbs using MRI has been actively reported [11,12]. Notably, according to the study investigating the morphology of the olfactory bulb in patients with olfactory dysfunction using MRI, it is known that the volume loss of the olfactory bulb progresses from a normal oval shape to a flattened shape. Consequently, a decrease in height is more pronounced compared to the width of the olfactory bulb [13]. Indeed, the studies comparing patients with olfactory dysfunction to healthy controls have shown that among 2D parameters, only height was statistically significant [11,13]. Furthermore, OBH has been identified as a predictive factor for the success of olfactory training in patients with olfactory loss [14].

Therefore, this study aimed to determine whether the OBH measured by MRI has clinical utility as an imaging biomarker for the evaluation of iPD patients through its correlation with movement impairment.

## Materials and methods

### Study design

This retrospective study was approved by the Institutional Review Board of Soonchunhyang University Bucheon Hospital, which waived the requirement for written informed consent (IRB No. SCHBC 2023-09-002). From August 2019 to November 2022, we recruited participants who met the selection criteria from among all consecutive patients who visited our neurology department movement disorders clinic and underwent brain MRI for suspected parkinsonism. The inclusion criteria were as follows: (a) age 18 years or older; and (b) both brain MRI and N-(3-fluoropropyl)-2β-carboxymethoxy-3β-(4-iodophenyl) nortropane (FP-CIT) positron emission tomography-computed tomography (PET-CT) were performed to evaluate parkinsonism. Participants were excluded if (a) the final clinical diagnosis was suspected to be atypical parkinsonism (progressive supranuclear palsy, multiple systemic atrophy, and corticobasal degeneration); (b) they had mild or more severe cognitive impairment; (c) they had ongoing neurological diseases other than parkinsonism; (d) the final clinical diagnosis was not obtained because of interruption of follow-up due to transfer, etc.; (e) there were structural abnormalities in the basal ganglia due to local brain lesions such as stroke, cerebral hemorrhage, etc.; (f) there was a local pathologic lesion in the olfactory bulb due to traumatic brain injury or infection; and (g) it was difficult to properly interpret the obtained MR image for technical reasons, such as motion artifacts. High-resolution T2-weighted imaging (T2WI) added to evaluate the olfactory bulb is noninvasive and does not require additional contrast media. Therefore, images were obtained after verbal informed consent was given to the participants for up to 5 min added to the MRI examination time. The research-purpose data, which were obtained from October 7, 2023 to December 31, 2023 were fully anonymized to eliminate any traces of personal information. The researchers did not have access to information that could identify individual participants during or after data collection.

### Clinical data acquisition

The final clinical diagnosis was determined by our attending neurologist (J.H.P, with 25 years of experience) based on clinical data, including medical records, MRI, and FP-CIT PET findings collected until August 2023. iPD was diagnosed according to the British Parkinson’s Disease Society Brain Bank Diagnostic Criteria, and the severity of motor symptoms was measured using the Unified Parkinson’s Disease Rating Scale (UPDRS) III and modified Hoehn and Yahr staging scale (HY). Among the measured results, time off levodopa 24 h, closest to the time of MRI, was recorded. The clinical laterality of motor symptoms was assessed by evaluating the UPDRS III scores. If the score of one side was two points higher than that of the other side, the symptom was defined as asymmetric [15]. Clinical olfactory function screening was performed using the order identification test of the Korean Version of the Sniffin’ Sticks II test, which uses 16 odors familiar to Koreans [16]. Testing was performed in the otolaryngology department by an experienced specialist after the otolaryngologist ruled out olfactory dysfunction due to a secondary etiology. Total scores range from 0 to 16, and scores of 11 to 16 are defined as “normosomia,” 0–10 as “decreased olfaction.” As there was an overlap with the coronavirus disease (COVID-19) pandemic period during the study, the time when participants underwent MRI was also recorded and analyzed.

### Image acquisition

All MR images were obtained using 3-T scanners (MAGNETOM Vida and MAGNETOM Skyra; Siemens Healthcare, Erlangen, Germany) with a 64-channel head coil. Patients were allocated randomly according to the MRI room timetable. Coronal 2-dimensional (D) turbo spin echo T2WI was used to evaluate the olfactory bulb. The detailed MR parameters included the following: matrix, 384 x 384; TR/TE, 3810/73 ms; number of averages, 2; FOV, 180 × 180 mm; section thickness, 1 mm without interval; voxel size, 0.43 × 0.43 × 1.5 mm; bandwidth, 407 Hz/pixel; scan time, 4.5 min.

### Image analysis

**Fig 1:**
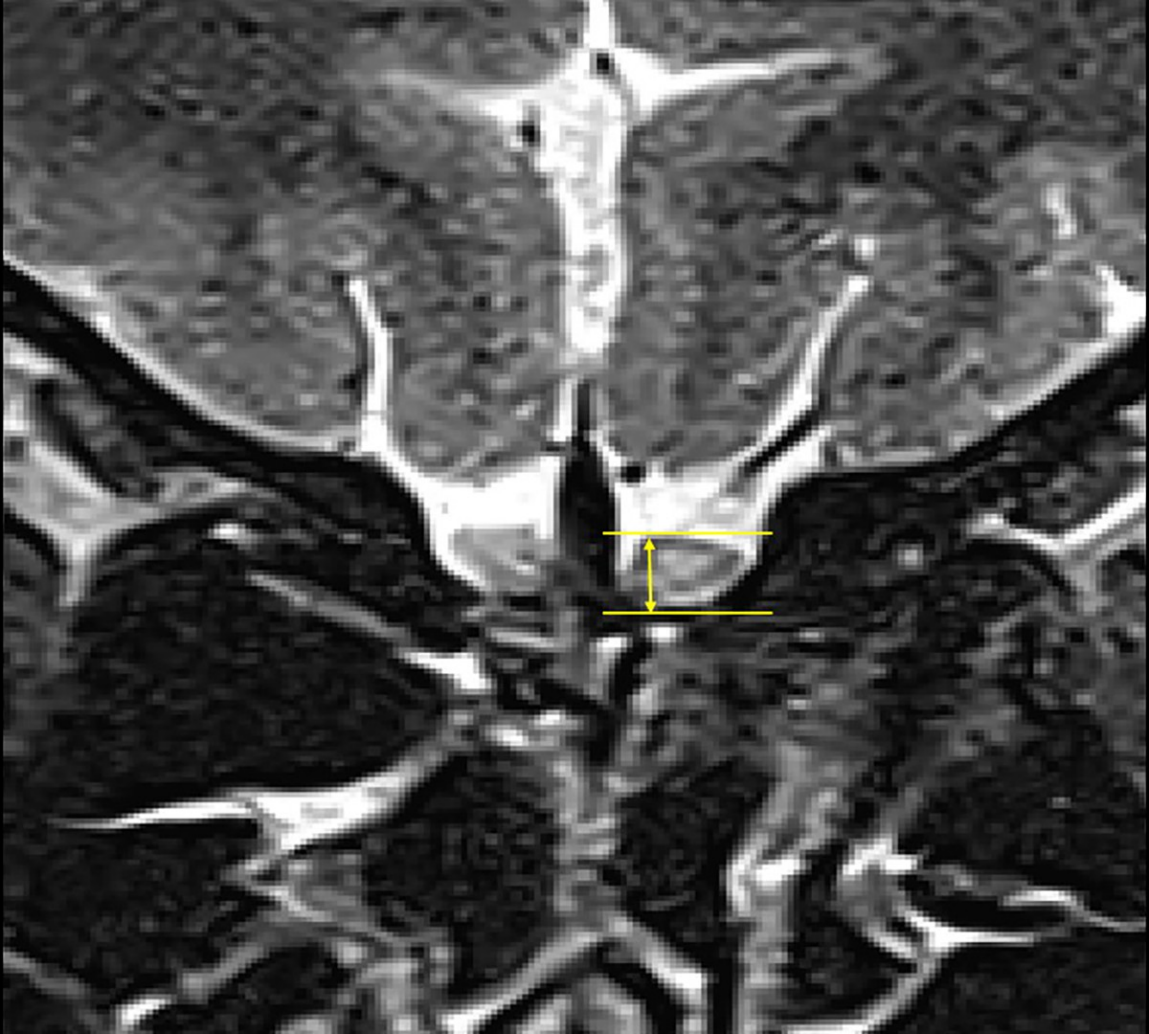
How to measure. A 31-year-old female patient who was diagnosed with drug-induced parkinsonism. Coronal T2-weighted imaging was used to measure olfactory bulb height.

Two board-certified neuroradiologists (J.B.L and J.Y.K, with 12 and 8 years of experience, respectively) independently evaluated the MR images and measured the OBH on 2D T2WI. Measurements were performed without knowledge of the patients’ clinical information. The measurement of OBH was underwent through manual measurement using in-house software (Deja view, Soonchunhyang University Bucheon hospital PACS, Bucheon, Korea). The image reviewer selected the image in which each OBH appeared largest among coronal T2WI and then used the zoom-in function (digital zoom without software image correction) of the software. At this time, the selected image was enlarged within a range 3 to 5 times [13] that did not cause image degradation at the margin of the olfactory bulb and then the OBH was measured. To avoid overestimation, if there was an overlap of structures, such as the olfactory bulb showing an inverted J shape, it was measured except for the part showing cerebrospinal fluid signal intensity (**Fig 1**).

### Statistical analysis

To compare the clinical data and OBH of the iPD and disease control groups, continuous variables were examined for normality using the Shapiro–Wilk test and then analyzed using the Mann–Whitney test or independent *t*-test according to each result. Nominal variables were compared using the chi-squared test. Univariable and multivariate logistic regression were used in the risk factor analysis of iPD, and age and sex, which are known to affect the OBH, were adjusted for. Subsequently, the optimal cutoff value was calculated using receiver operating characteristic (ROC) analysis and the Youden index. Based on the laterality of motor symptoms, the Kruskal-Wallis test was employed to compare the OBH of the ipsilateral, contralateral side, and the OBH of disease control group. Post-hoc analysis was subsequently conducted using Dunn’s method. One-way Analysis of Variance was used to analyze whether there was a significant difference in OBH according to the HY scale and year of MRI. If there was a significant difference, the Scheffé post hoc test was used. The correlation between UPDRS III and OBH was calculated using Pearson’s correlation, and the calculated r values were classified as follows: 0–0.3 = poor, 0.3–0.6 = fair, 0.6–0.8 = good, and 0.8–1 = very strong [17]. The interobserver agreement was calculated using the intraclass correlation coefficient (ICC), and a two-way mixed model single measure was used. The ICC values were classified as follows: <0.5 = poor, 0.50–0.75 = moderate, 0.75–0.90 = good, and >90 = excellent [18]. Statistical analyses were performed using R statistical software version 3.6.1; The R Foundation for Statistical Computing, Vienna, Austria) and MedCalc version 22.016 (MedCalc Software, Ostend, Belgium). Statistical significance was set at *p* value less than 0.05.

## Results

### Study participants

**Fig 2:**
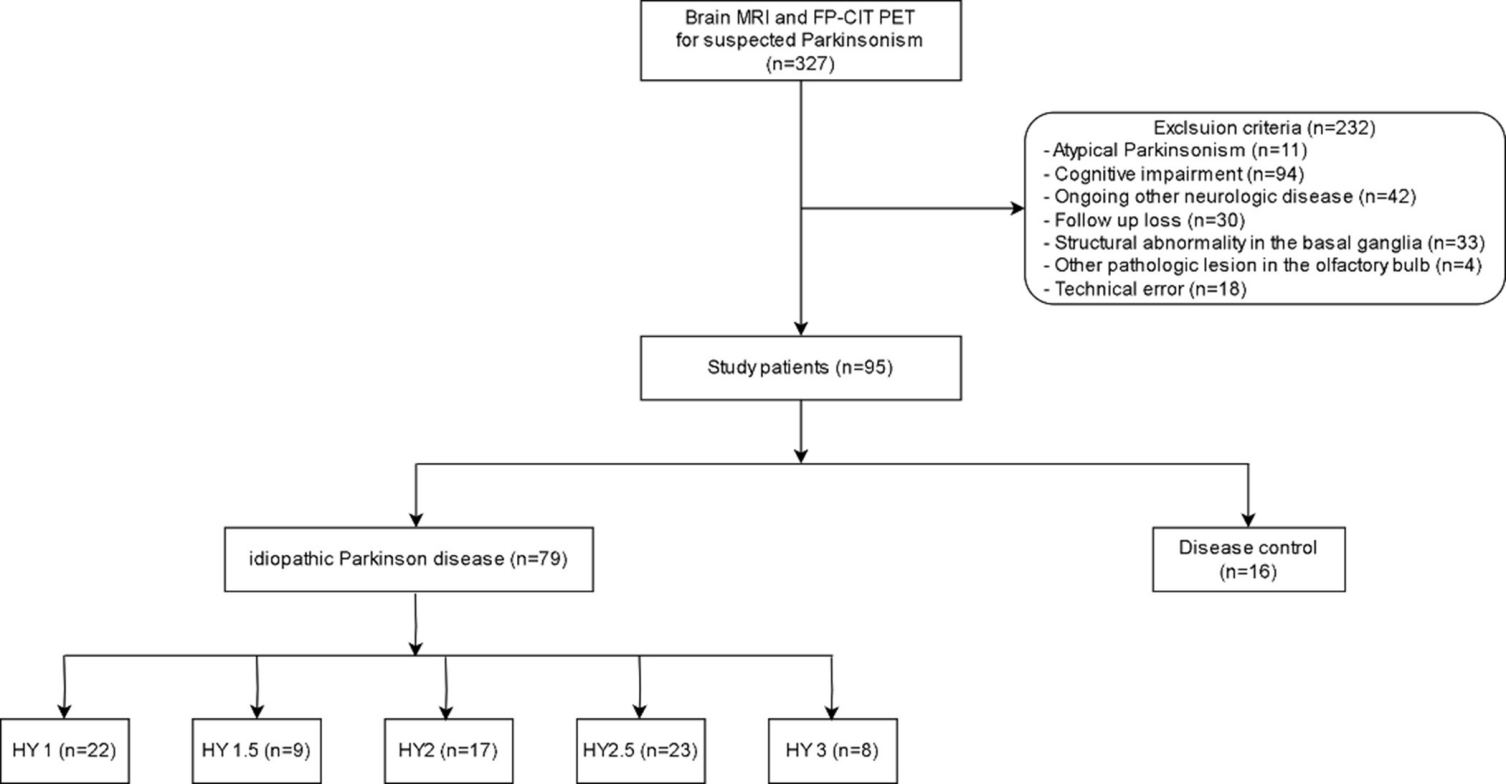
Patient flow diagram. MRI, magnetic resonance imaging; FP-CIT, N-3-fluoropropyl-2-carbomethoxy-3-4iodophenyl nortropane; PET, positron emission tomography; HY1-3, modified Hoehn and Yahr scale stage 1–3.

From August 2019 to November 2022, 327 participants underwent brain MRI and FP-CIT PET-CT to evaluate parkinsonism. Of these, 232 participants were excluded for the following reasons: atypical parkinsonism (*n* = 11), cognitive impairment (*n* = 94), other ongoing neurological diseases (*n* = 42), follow-up loss (*n* = 30), structural abnormalities in the basal ganglia (*n* = 33), other pathological lesions in the OB (*n* = 4), and technical errors (*n* = 18). A total of 95 consecutive patients were included in the analysis (**Fig 2**), and their demographic and clinical data are summarized in Table 1. A total of 79 patients were diagnosed with iPD based on the final clinical diagnosis, and 16 patients were assigned to the disease control group. The disease control group defined as the patients who exhibiting movement disorders extrinsic to nigrostriatal dopaminergic degeneration, such as essential tremor, drug-induced Parkinsonism, or etc. These patients initially showed ambiguous Parkinsonism symptoms but demonstrated normal FP-CIT PET findings and the diagnosis solidified after clinical follow up.

**Table 1:** Demographics of the study population.

Characteristic	iPD (*n* = 79)	Disease control (*n* = 16)	*p* value
Age	67.0 (60.3–72.0)	75.0 (64.5–79.0)	0.07^a^
Sex			> 0.05^b^
Male	42	8	
Female	37	8	
Disease duration	6 (3.13–17.3)	42 (12.0–138.0)	< 0.05^a^
UPDRS III score (OFF state)	19 (13–28)^d^	^f^NA	
Clinical motor asymmetry	58	^f^NA	
Right	40	^f^NA	
Left	18	^f^NA	
HY stage (OFF state)	2 (1–2.5)^d^	^f^NA	
1	22		
1.5	9		
2	17		
2.5	23		
3	8		
OBH (mm)	1.42 ± 0.19	1.73 ± 0.21	< 0.0001^c^
Decreased olfaction		^f^NA	
Normal	62		
Decreased	17		
Study year			
2019	8	0	
2020	24	6	
2021	21	3	
2022	26	7	

Data are presented as median (interquartile range), the number of patients, or mean ± standard deviation.

^a^Disease control group was defined as patients exhibiting movement disorders unrelated to nigrostriatal degeneration.

^b^ *p* values were calculated using the Mann–Whitney test.

^c^ *p* value was calculated using chi-squared test.

^d^ Among continuous variables, if equal variance and normal distribution were satisfied, an independent t-test was performed to obtain a *p* value.

^e^ Seventeen patients were excluded because their OFF-state HY stage and UPDRS III scores were not available in their electronic medical records.

^F^NA, in the disease control group, the data related to UPDRS III, HY stage, motor asymmetry, and KVSS test (decreased olfaction) are not available (NA)

*iPD*, idiopathic Parkinson disease; *UPDRS III*, Unified Parkinson’s Disease Rating Scale part III; *HY*, Hoehn and Yahr; *OBH*, olfactory bulb height.

There were no significant differences in age or sex between the two groups. Clinical motor asymmetry was observed in 58/79 patients in the iPD group (n; Rt/Lt: 40/18). The HY stage of the OFF state was measured in 22, 9, 17, 23, and 8 iPD patients as HY 1, 1.5, 2, 2.5, and 3, respectively. From 2019 to 2022, the number of patients who underwent MRI by year was 8, 24, 21, and 26 in the iPD group and 0, 6, 3, and 7 in the disease control group, respectively.

### Risk factor analysis in iPD vs. disease control

**Fig 3:**
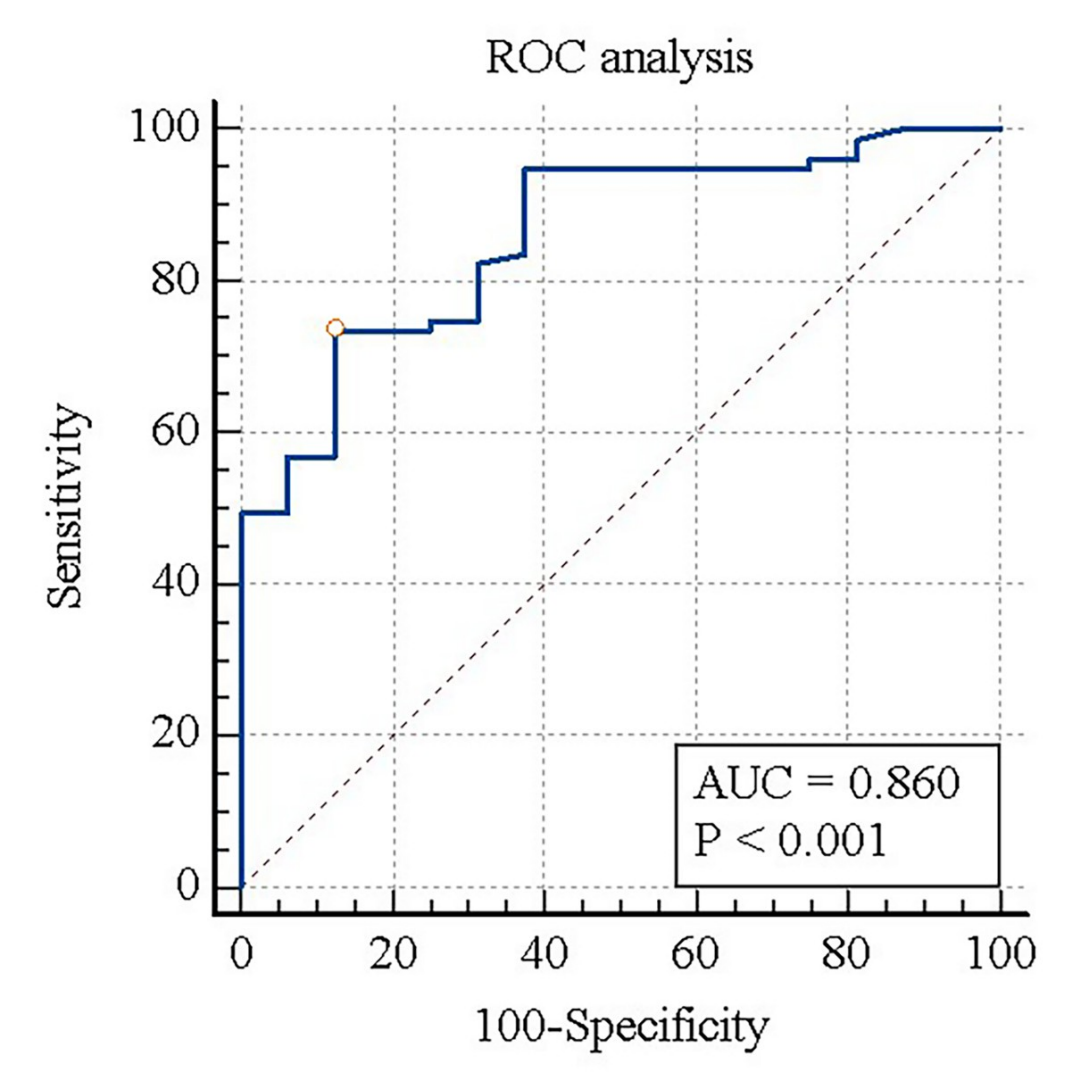
Receiver operating characteristic curve for idiopathic Parkinson’s disease discrimination based on olfactory bulb height. The circle represents the cutoff point calculated by Youden index (≤ 1.52 mm, Sensitivity: 73.4%, Specificity: 87.5%). ROC, receiver operating characteristic curve; AUC, area under the curve.

The OBH was 1.42 mm ± 0.19 (standard deviation [SD]) in the iPD group and 1.73 mm ± 0.21 (SD) in the disease control group, and was statistically significantly smaller in the iPD group (*p* < 0.0001) (**Table 1 and Fig 2**). The inter-observer agreement of the olfactory bulb using ICC was good (0.86, 95% confidence interval [CI]: 0.82–0.90). In all participants, when risk factor analysis was performed using univariable logistic regression, OBH was a statistically significant risk factor distinguishing the iPD group from the disease control group (odds ratio [OR] 0.0012, 95% CI: 0.0002–0.0315). Additionally, when multivariate logistic regression was performed after adjusting for age and sex, which are other factors affecting olfactory bulb volume [19], OBH was found to be a statistically significant independent risk factor (OR 0.0003, 95% CI: 0.0000–0.0129) (**Table 2**). In the diagnostic performance test using the ROC curve, the area under the curve for distinguishing iPD of OBH from disease control was 0.86 (95% CI: 0.77–0.92), and the optimal cutoff by the Youden index was 1.52 mm (sensitivity 73.4%, specificity 87.5%) (**Fig 3**).

**Table 2:** Risk factor analysis in idiopathic Parkinson’s disease patients vs. disease controls.

Variable	Univariable		Adjusted model (age and sex)	
	OR (95% CI)	*p* value	OR (95% CI)	*p* value
Age	0.9593 (0.9047–1.0172)	0.1643	0.9106 (0.8468–0.9793)	0.0116
Sex	0.8810 (0.3007–2.5813)	0.8172	0.8099 (0.2031–3.2302)	0.7652
OBH (mm)	0.0012 (0.0002–0.0315)	0.0001	0.0003 (0.0000–0.0129)	< 0.0001

*OR*, odds ratio; *CI*, confidence interval; *OBH*, olfactory bulb height.

### Comparison of OBH according to clinical motor asymmetry in iPD

The OBH measured in iPD patients with clinical motor asymmetry (n = 58) was significantly smaller on both the ipsilateral and contralateral sides of motor asymmetry compared to the those of disease control group. However, there was no significant difference between the ipsilateral and contralateral sides within the iPD group (Median [interquartile range]; iPD with clinical motor asymmetry, ipsilateral: 1.43mm [1.28–1.53]; contralateral: 1.44mm [1.25–1.53]; disease control, right: 1.77mm [1.61–1.86; left: 1.69mm [1.56–1.85], *P* < 0.05, **Table 3** and **S1 Fig**). In comparisons of each right and left sides between the group with iPD and the disease control group, both sides of the iPD group with motor asymmetry showed significantly smaller OBH values on both sides of disease control (*P* < 0.05, **Table 3** and **S2 Fig**).

**Table 3:** 

Subjects		OBH (mm)	Different from (*P* < 0.05)
iPD with motor laterality	Ipsilateral (n = 58)	1.43 (1.28–1.53)	Disease control, all^a^
	Contralateral (n = 58)	1.44 (1.25–1.53)	Disease control, all^a^
	Right (n = 58)	1.41 (1.28–1.50)	Disese control, Right^b^
	Left (n = 58)	1.46 (1.25–1.54)	Disease control, Left^b^
Disease control	All (n = 32)	1.70 (1.60–1.86)	iPD with motor asymmetry, Ipsilateral^a^, iPD with motor asymmetry, Contralateral^a^
	Right (n = 16)	1.77 (1.61–1.86)	iPD, Right^b^
	Left (n = 16)	1.69 (1.56–1.85)	iPD. Left^b^

Data are presented as median (interquartile range).

^a^The Kruskal-Wallis test was used to compare the OBH of the ipsilateral, contralateral side, and the OBH of disease control group. Post-hoc analysis was subsequently conducted using Dunn’s method.

^b^Comparisons of the right and left sides between the disease control group and the iPD with motor asymmetry group were conducted using the Mann-Whitney test.

*iPD*, idiopathic Parkinson disease*; OBH*, olfactory bulb height.

### Correlation between OBH and motor symptom scales

**Fig 4:**
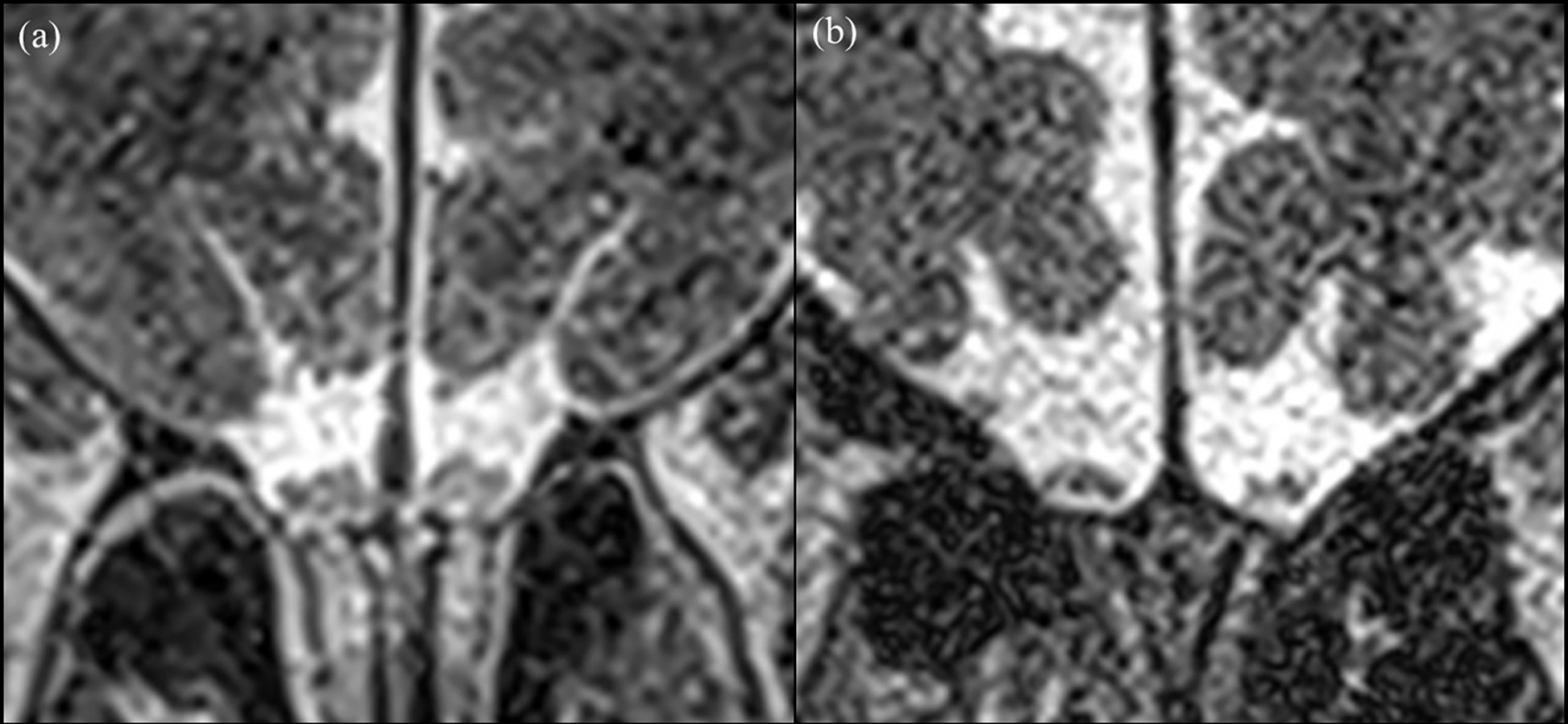
Representative cases. (a) Images obtained from a 64-year-old man with idiopathic Parkinson’s disease (iPD), with modified Hoehn and Yahr scale stage (HY) 1. On the coronal T2-weighted image, each left and right olfactory bulb was observed, and the average of olfactory bulb height (OBH) was 1.72 mm. (b) On the image of an 84-year-old female iPD patient with HY3, the measured OBH was 1.16 mm.

**Fig 5:**
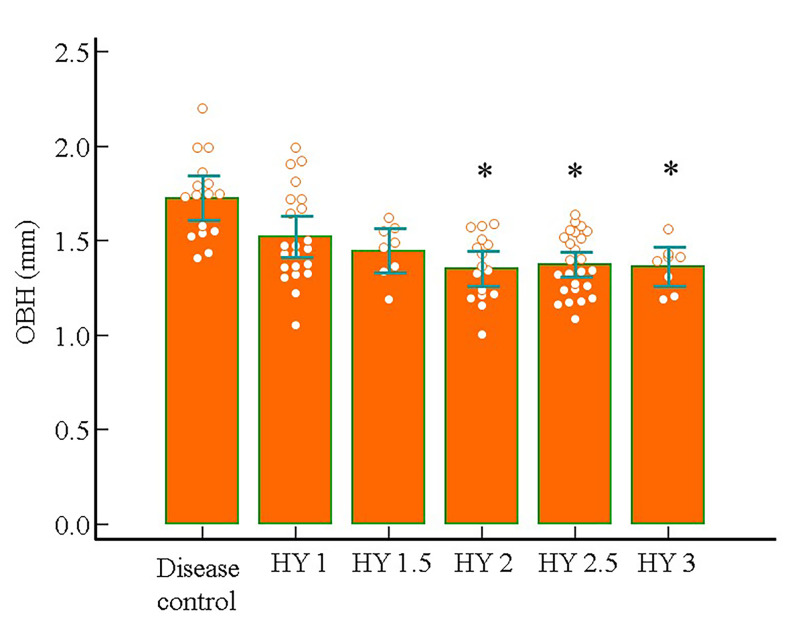
Olfactory bulb height according to the modified Hoehn and Yahr scale stage. Multiple comparison bar graph showing the olfactory bulb height (OBH) of the disease control and modified Hoehn and Yahr scale stage (HY) 1–3 groups. One-way analysis of variance revealed a significant difference in OBH between the groups (*p* < 0.05). Asterisk (*) indicates a statistically significant difference (*p* < 0.05) in disease control and post-hoc tests (Scheffe method).

**Fig 6:**
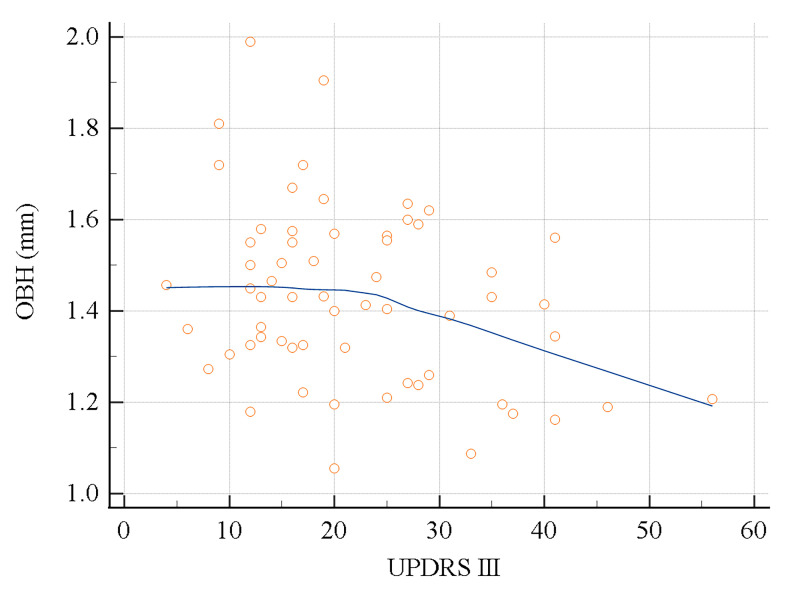
Correlation between olfactory bulb height and Unified Parkinson Disease Rating Scale III). Relationship between olfactory bulb height (OBH) and Unified Parkinson Disease Rating Scale III (UPDRS III) scores in the idiopathic Parkinson’s disease group (n = 62). The scatter plot shows a significant association between OBH and UPDRS III, where the higher the UPDRS III score, the smaller the OBH (r = -0.32, *p* = 0.0126).

In all participants (*n* = 95), OBH according to HY stage showed significant differences from that in the disease controls, in the HY 2 or higher group (disease control 1.73 ± 0.22 mm; mHY2 1.35 ± 0.17 mm; mHY2.5 1.37 ± 0.16 mm; HY3 1.36 ± 0.12 mm). The mHY1-1.5 group also had a smaller OBH compared to the disease controls (HY1 1.52 ± 0.24 mm; mHY1.5 1.45 ± 0.14 mm), but this was not statistically significant (**Table 4 and Fig 4**). **Fig 5** shows the representative MR images. In the iPD group, in which UPDRS III in the OFF state was available (*n* = 62), a negative correlation was observed between UPDRS III and OBH (*r* = -0.32, *p* = 0.0126) (**Fig 6**).

**Table 4:** The olfactory bulb height according to modified Hoehn and Yahr staging scale.

	Disease control (*n* = 16)	HY1 (*n* = 22)	HY1.5 (*n* = 8)	HY2 (*n* = 16)	HY2.5 (*n* = 25)	HY3 (*n* = 8)	*p* value^a^
Mean (mm)	1.73	1.52	1.45	1.35	1.37	1.36	< 0.001
SD (mm)	0.22	0.24	0.14	0.17	0.16	0.12	
Different (*p*^b^ < 0.05) from	HY2, HY2.5, HY3			Disease control	Disease control	Disease control	

^a^ *p* value was calculated using one-way analysis of variance (ANOVA).

^b^ *p* value was obtained by Scheffé post-hoc test.

*HY*, Hoehn and Yahr; *OBH*, olfactory bulb height; SD, standard deviation.

### Correlation between OBH and timing of imaging study

When comparing OBH according to the year in which MRI was performed in all participants, there was no statistically significant difference (*p* = 0.482) (**S3 Fig**).

## Discussion

In this study, OBH was a statistically significant independent risk factor for distinguishing iPD from the disease control group, and the optimal cutoff was 1.52 mm. In a subgroup analysis performed to evaluate the correlation with the laterality of motor symptoms, there was no significant difference in OBH between the ipsilateral and contralateral sides in the group with asymmetry in motor symptoms. However, significant correlations were observed with scales measuring the severity of motor symptoms. The comparison of OBH between the disease control group and iPD group according to HY stage showed a statistically significant difference at iPD with HY2 or higher. In the correlation evaluation between OBH and UPDRS III performed in iPD patients, OBH and UPDRS III showed a negative correlation.

The results of previous studies of MR-measured olfactory bulb atrophy in patients with iPD vary. In a study evaluating olfactory bulb atrophy in iPD using 3T MRI, Brodoehl et al. [7] found that the olfactory bulb volume and height measured on heavy T2WI were significantly smaller in iPD patients than in healthy controls. In a study by Paschen et al. [9], which was later conducted with more participants, the volume was measured using coronal 2D T2WI, but no significant difference was noted. A meta-analysis of six studies reported that the olfactory bulb volume of iPD patients was significantly smaller than healthy controls [8]. Additionally, recent studies on the correlation between olfactory function and the severity of motor symptoms have reported that a decrease in olfactory function appears in the relatively early stages of iPD. The results of the present study are consistent with those of the aforementioned studies. When analyzing the entire participants, the OBH measured in the iPD group was significantly smaller than that in the disease control group. However, in the subgroup analysis, the mHY1-1.5 group showed no significant difference from the disease control group. Therefore, one hypothesis is that olfactory atrophy in iPD may not be clearly reflected if the study group includes a relatively large number of early-stage iPD patients. Notably, when comparing studies that did not show a significant difference in olfactory volume to those that did, the HY scale score of the latter was relatively high. This tendency to underestimate olfactory bulb volume loss in the relatively early stages of the disease is similar to the results of studies not only in iPD but also in Alzheimer’s disease, where olfactory volume loss becomes more severe as the dementia stage progresses [19]. Considering that olfactory dysfunction has also been reported in many other neurodegenerative diseases such as Lewy body disease, and cognitive impairment [20], it is possible that olfactory bulb volume loss may not be evident in the early stages of these diseases. Therefore, when planning future research to investigate the relationship between neurodegenerative diseases and olfactory bulb atrophy, researchers should be mindful that the loss of olfactory bulb volume may not be sufficiently reflected in the early stages of the disease.

Our study is clinically significant because of the following features: First, it was possible to obtain an optimal cutoff that divided the iPD and disease control groups by analyzing the data obtained while evaluating parkinsonism. Considering that the cutoff calculated in this study was 1.52 mm, there was an overlap of 95% CI up to the mHY2 group; therefore, early diagnosis seems to be less useful. In addition, because cognitive impairment is known to affect olfactory bulb atrophy [21], the results of this study only targeted cognitively intact iPD, and taking this into consideration, it is estimated that the clinical usefulness in neurodegenerative disorder evaluation situations will be further reduced. Second, there was no significant difference between the ipsilateral and contralateral sides in the comparison of motor asymmetry, which suggests that the observed olfactory bulb asymmetry may not be due to the involvement of iPD but other causes. Finally, the results showing a correlation between the HY scale and UPDRS III indicate the possibility that the OBH can be used as a tool to evaluate the disease progression of iPD. Currently, tools used to evaluate disease progression in imaging studies are focused on assessing the dopaminergic pathway [22]. According to Braak’s hypothesis, OBH has the advantage of evaluating anatomically different structures involved at an earlier stage [3]. Therefore, OBH has the potential to be used as an additional measure to monitor disease progression in the early stage and can be used as a basis for screening olfactory function in the evaluation of iPD patients and further evaluating it when abnormalities are found.

Despite its merits, this study has several limitations. First, only OBH was included in the analysis, and data such as olfactory length, width, and volume were not obtained. This was a retrospective pilot study, and since the purpose of the MRI examination was parkinsonism evaluation, it was designed to obtain 2D images instead of 3D volumetric images, which require a relatively long scan time. Using a 2D image allows the signal intensity of the T2WI of the olfactory bulb to be checked, allowing for pathologies other than atrophy to be screened, and a single measurement at the maximum length is advantageous in terms of interobserver agreement compared to the volume that requires three measurements. The OBH measurement in this study obtained high ICC values, similar to those in a previous study [11]. However, with recent developments in AI and MRI technology, it is also possible to analyze differences in the morphology or volume of the olfactory bulb using AI [23,24]. Therefore, the results of this study should be confirmed in future large-scale studies using these resources. Second, this study overlapped with the COVID-19 pandemic. There are reports that there may be an olfactory bulb volume change in COVID-19 [25], and this may have influenced the study results. However, this institution applied a strict protocol to confirm a negative COVID-19 polymerase chain reaction test result before patients entered the hospital during the pandemic period, confirmed the signal intensity on T2WI, and evaluated the difference in OBH by year, showing no significant difference (**S3 Fig**). Therefore, it is assumed that COVID-19 will have little impact on research. Third, in the case of the disease control group, some clinical data, including clinical olfactory function screening, were not obtained. This is a limitation of the retrospective study design, and due to not only there was no medical rationale for recommending additional costly and time-consuming tests for clinically asymptomatic patients, but also we were unable to obtain patient consent. Given that the incidence of olfactory dysfunction as measured by objective assessment is 2.7–24.5% [20], the measured olfactory height in the disease control group of this study may have been underestimated compared to healthy subjects.

In conclusion, OBH is decreased in iPD patients regardless of age and sex and may be correlated with the progression of motor symptoms in iPD. Therefore, OBH measured using MRI has the potential to be used to evaluate early-stage disease progression in iPD patients.

## Supporting information

S1 Fig OBH, olfactory bulb height. (DOCX)

S2 Fig iPD, idiopathic Parkinson disease. (DOCX)

S3 Fig OBH, olfactory bulb height; MRI, magnetic resonance imaging. (DOCX)
