# Supplementary material for: Clinical significance of MRI-measured olfactory bulb height as an imaging biomarker of idiopathic Parkinson’s disease
Source: PLoS One. 2024 Oct 28;19(10):e0312728. doi: 10.1371/journal.pone.0312728 (PMC11515979; doi:10.1371/journal.pone.0312728)
Supplement: S1 Fig — (DOCX) [file pone.0312728.s001.docx]

**ELECTRONIC SUPPLEMENTARY MATERIAL**


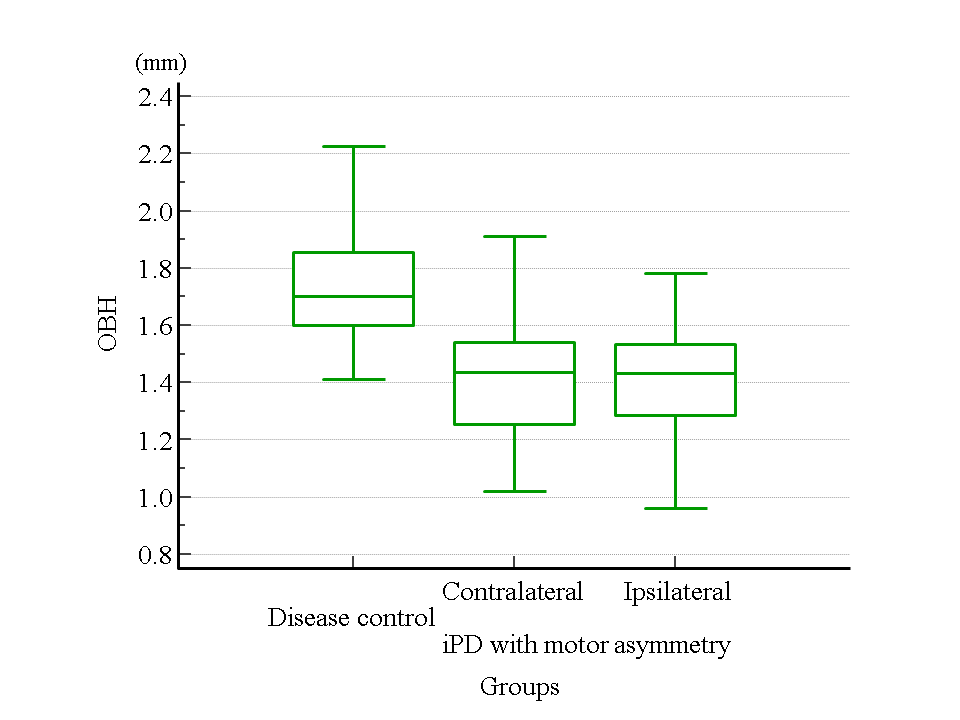
**S1 Fig. Comparison of olfactory bulb height according to clinical laterality**
