# Supplementary material for: Clinical significance of MRI-measured olfactory bulb height as an imaging biomarker of idiopathic Parkinson’s disease
Source: PLoS One. 2024 Oct 28;19(10):e0312728. doi: 10.1371/journal.pone.0312728 (PMC11515979; doi:10.1371/journal.pone.0312728)
Supplement: S2 Fig — (DOCX) [file pone.0312728.s002.docx]

**ELECTRONIC SUPPLEMENTARY MATERIAL**

**S2 Fig. Comparison of each side of olfactory bulb height (disease control group vs idiopathic Parkinson disease with motor asymmetry group)**

**
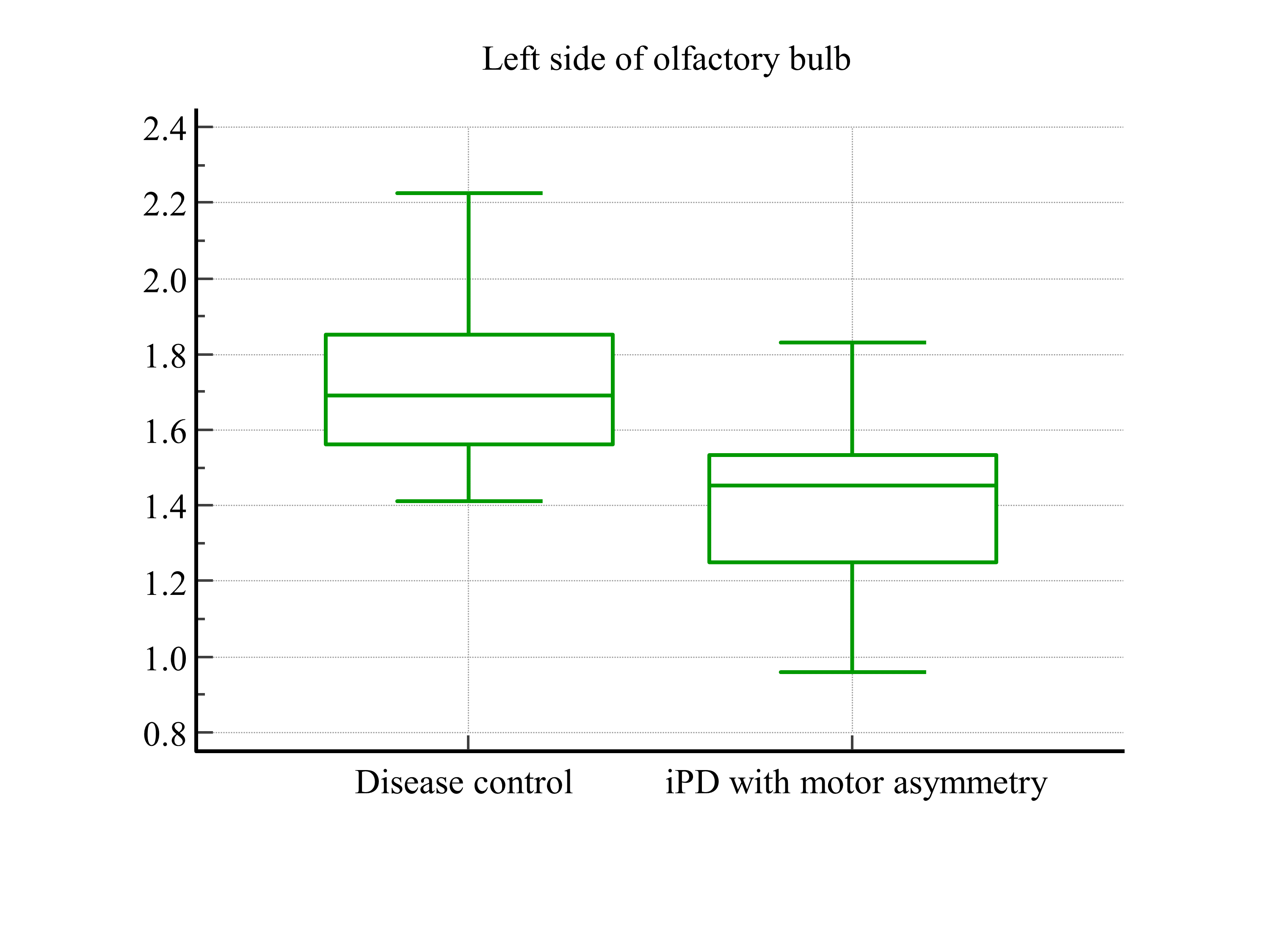

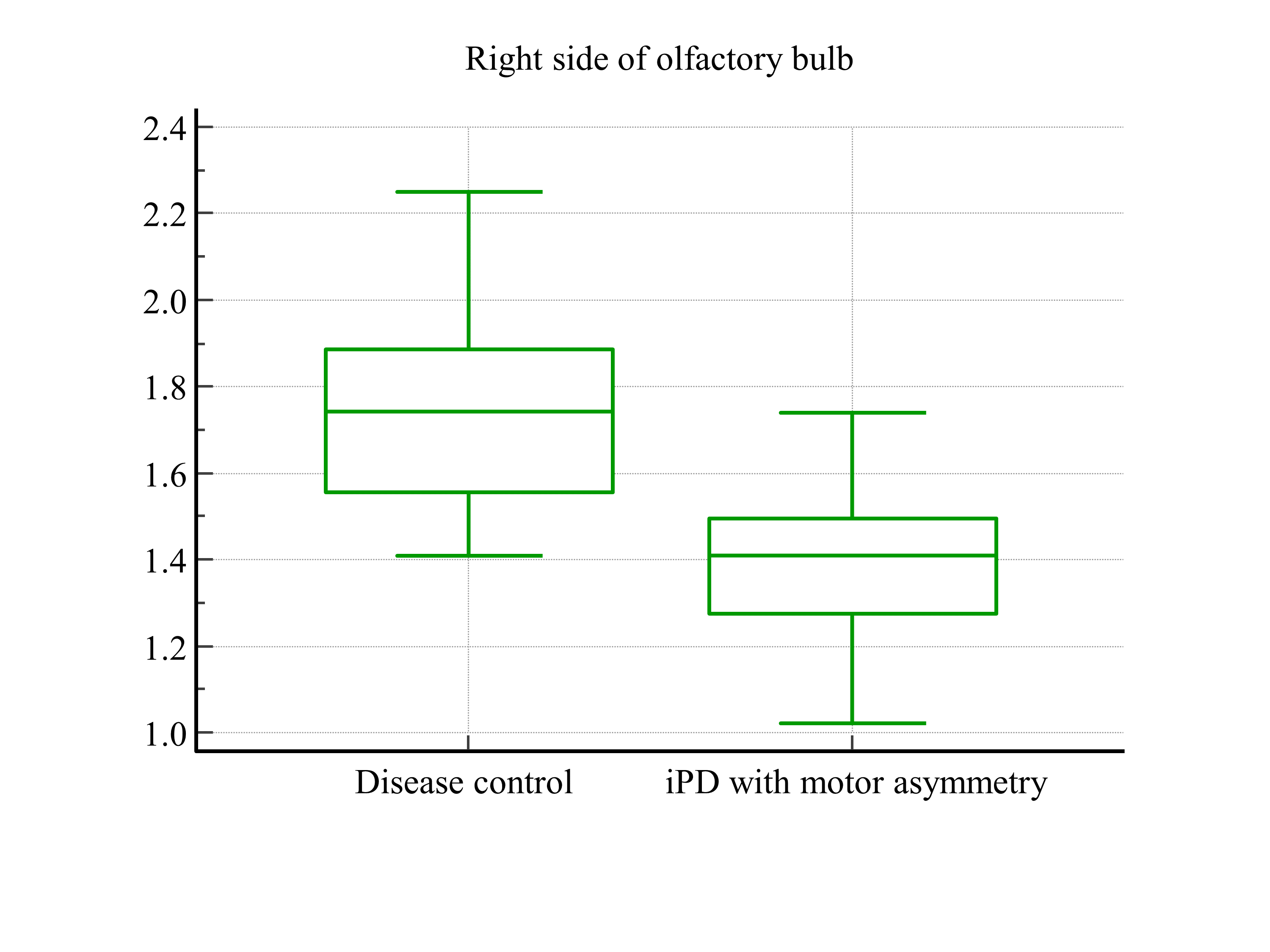
**
