# Supplementary figures and images for: Clinical significance of MRI-measured olfactory bulb height as an imaging biomarker of idiopathic Parkinson’s disease
Source: PLoS One. 2024 Oct 28;19(10):e0312728. doi: 10.1371/journal.pone.0312728 (PMC11515979; doi:10.1371/journal.pone.0312728)

**ELECTRONIC SUPPLEMENTARY MATERIAL**

**S3 Fig. Correlation between OBH and timing of imaging study**


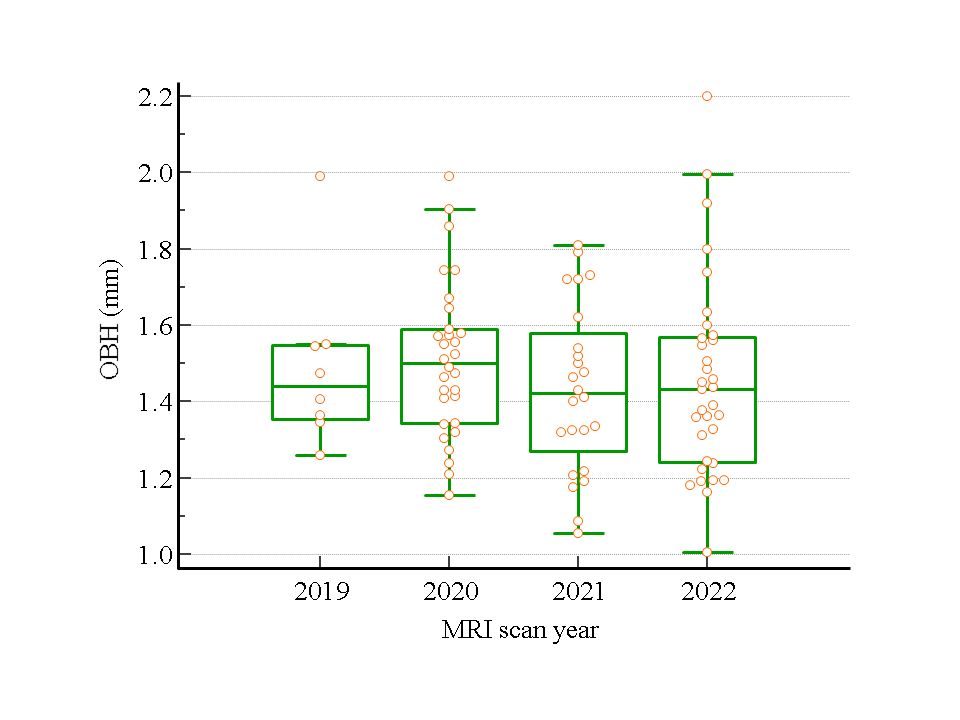

Supplement: S3 Fig — (DOCX) [file pone.0312728.s003.docx]
